# The Impact of Recombination on dN/dS within Recently Emerged Bacterial Clones

**DOI:** 10.1371/journal.ppat.1002129

**Published:** 2011-07-14

**Authors:** Santiago Castillo-Ramírez, Simon R. Harris, Matthew T. G. Holden, Miao He, Julian Parkhill, Stephen D. Bentley, Edward J. Feil

**Affiliations:** 1 Department of Biology and Biochemistry, University of Bath, Claverton Down, Bath, United Kingdom; 2 The Wellcome Trust Sanger Institute, Wellcome Trust Genome Campus, Hinxton, Cambridge, United Kingdom; Imperial College, United Kingdom

## Abstract

The development of next-generation sequencing platforms is set to reveal an unprecedented level of detail on short-term molecular evolutionary processes in bacteria. Here we re-analyse genome-wide single nucleotide polymorphism (SNP) datasets for recently emerged clones of methicillin resistant *Staphylococcus aureus* (MRSA) and *Clostridium difficile*. We note a highly significant enrichment of synonymous SNPs in those genes which have been affected by recombination, i.e. those genes on mobile elements designated “non-core” (in the case of *S. aureus*), or those core genes which have been affected by homologous replacements (*S. aureus* and *C. difficile*). This observation suggests that the previously documented decrease in dN/dS over time in bacteria applies not only to genomes of differing levels of divergence overall, but also to horizontally acquired genes of differing levels of divergence within a single genome. We also consider the role of increased drift acting on recently emerged, highly specialised clones, and the impact of recombination on selection at linked sites. This work has implications for a wide range of genomic analyses.

## Author Summary

As bacteria diversify, many of the nucleotide changes that emerge will render the cell slightly less competitive, and these mutations will tend to be removed by natural selection. However, this purging process does not happen instantaneously, and this delay allows deleterious mutations to survive in the population long enough to be sampled. Genomes at the very initial stages of diversification therefore exhibit a relatively high proportion of slightly deleterious mutations and, as most of these are non-synonymous mutations, this is manifest as a high dN/dS ratio. However, the effective population size will also impact on this ratio, as will selection operating on neighbouring SNPs. Here we examine the distribution of synonymous and non-synonymous SNPs within recently emerged clones of two important nosocomial pathogens, methicillin resistant *Staphylococcus aureus* (MRSA) and *Clostridium difficile*. In both species, we note a much higher proportion of synonymous changes in those single nucleotide polymorphisms (SNPs) likely to have emerged through recombination compared to *de novo* mutations. We argue that this effect is explained by the very recent emergence of the mutational SNPs combined with a reduction in the efficiency of selection due to niche specialisation.

## Introduction

The populations of many pathogenic bacterial species exist as a collection of discrete clonal complexes, many of which have emerged recently and exhibit specific resistance or virulence attributes. For example, molecular techniques such as multilocus sequence typing (MLST) [1] have identified a small number of widely disseminated methicillin resistant *S. aureus* (MRSA) clonal lineages [2], [3]. Of these, the MLST haplotype Sequence Type (ST) 239 is the most common globally [4], [5], [6], [7], [8], [9], [10]. This clone is multiple antibiotic resistant, shows increased virulence [11], [12], and is known to have emerged through a very large (635 kb) homologous replacement via an unknown mechanism [13], [14], [15]. Harris et al recently sequenced a global sample of 63 isolates of this clone using the Illumina Genome Analyser (IGA) platform [16]. Read mapping was carried out against a completely sequenced ST239 isolate (TW20) which caused an outbreak in a London hospital [11] [14], and this approach yielded powerful evidence concerning the global dissemination of this clone. Comparable studies using the Illumina platform have subsequently been carried out on single clones in other pathogenic species, including *Streptococcus pneumoniae* [17] and *Clostridium difficile* [18].

Robust phylogenetic and evolutionary analysis on many bacterial genomes, and *S. aureus* in particular, relies on drawing a clear distinction between the “core” and “non-core” genomes [19], [20], [21], [22], [23]. The *S. aureus* core genome is stable, with modest levels of sequence divergence (typically ∼1.5%), low rates of homologous recombination, and a high degree of synteny [24], [25], [26]. In contrast, the non-core genome is highly diverse and dynamic due to high rates of horizontal transfer of accessory elements, including a number of genomic islands, various “types” of the SCC*mec* chromosomal antibiotic resistance cassette, and prophages encoding various toxins and other virulence attributes [25], [27], [28]. Harris et al defined core SNPs conservatively as those affecting contiguous regions (>1 kb) that were universally present within all the ST239 isolates sequenced [16]. A very low rate of homoplasy confirmed that the vast majority of the core SNPs arose by recent *de novo* mutation, rather than recombination. Analysis of the core data also suggested that ST239 first emerged in the mid 1960s, shortly after the first administration of methicillin in 1959.

Intuitively, it might be expected that non-core genes will exhibit a higher proportion of non-synonymous change than core genes. As non-core genes are not ubiquitous and therefore - by definition - non-essential, they might be under weaker selective constraints. Additionally, non-core genes encoding virulence factors may be subject to positive selection from the immune response [29]. However, non-core genes are typically acquired by horizontal transfer, and tend to exhibit striking mosaic structure, implicating a long history of recombination [23], [30], [31], [32], [33]. It follows that the *de novo* (mutational) emergence of many SNPs in the non-core may pre-date the emergence of the clonal lineage in which they are observed, particularly so if the clone has emerged very recently and spread very rapidly, as is the case with ST239. The age of the SNPs is important because very recently emerged *de novo* mutations tend to contain a high proportion of non-synonymous changes, not as a result of positive selection but because purifying selection has had insufficient time to purge slightly deleterious mutations [34], [35], [36], [37], [38]. Whilst this effect might be pronounced for recently emerged SNPs in the core genes, many of the non-core SNPs will have already passed through a selective filter in the wider population prior to transfer and so should show a lower proportion of slightly deleterious (non-synonymous) changes.

An important caveat of this time-dependence model is that the relative enrichment of non-synonymous changes within mutational SNPs should only be apparent when comparisons are made between very closely related isolates belonging to the same recently emerged clone, and recombination should have little impact on dN/dS when more diverged strains of the species are considered. Furthermore, core genes which have experienced homologous replacement originating from an external lineage should, like non-core genes, also show an enrichment of synonymous change. This provides the means to test alternative explanations which assume differing selective pressures acting on core and non-core genes.

In addition to the age of the SNPs, it is also necessary to consider variation in the efficiency of selection, at both a genome-wide level and at linked sites. For example, the adoption of a highly specialised niche would decrease the effective population size leading to greater drift (higher dN/dS). This would be more pronounced in the core relative to the mobile non-core, as the latter has been acquired horizontally from the wider population. Local rates of recombination also impact on the degree to which selection removes linked neutral variation [39], [40]. Regardless of whether selection is positive (hitch hiking [41]) or negative (background selection [42]) a greater degree of linked neutral variation will be removed in lowly recombining regions. This might also account for differences in dN/dS between core (non-recombining) and non-core (recombining) components of the genome. Unlike the time dependence model, there is no obvious reason why these effects should be restricted to very short evolutionary time-scales (within single clones).

Here we revisit the genome-wide ST239 SNP data for *S. aureus* [16] and *C. difficile* [18] to examine the impact of recombination on the proportion of synonymous and non-syonymous SNPs. For *S. aureus*, we confirm a higher rate of recombination and a highly significant enrichment of synonymous changes in the non-core. Further, we note a striking time dependence of dN/dS in non-core genes, whereby the more diverged elements (older SNPs) correspond to lower dN/dS ratios. However, our results also point to a moderation of the efficiency of purifying selection (i.e. increased drift) in core genes of *S. aureus* ST239, possibly owing to ecological specialisation and a reduction in the effective population size. This difference between core and non-core is not evident when more diverged (interclonal) comparisons are made, which argues against a strong effect from selection at linked sites. We note a similar enrichment of synonymous SNPs in core genes which have experienced homologous replacement, both in *S. aureus* and in *Clostridium difficile*. This confirms that the effect is not limited to *S. aureus*, and controls for the possibility of differing selection pressures acting on the core and non-core.

## Results

### SNP density and recombination in *S. aureus* ST239

**Figure 1:**
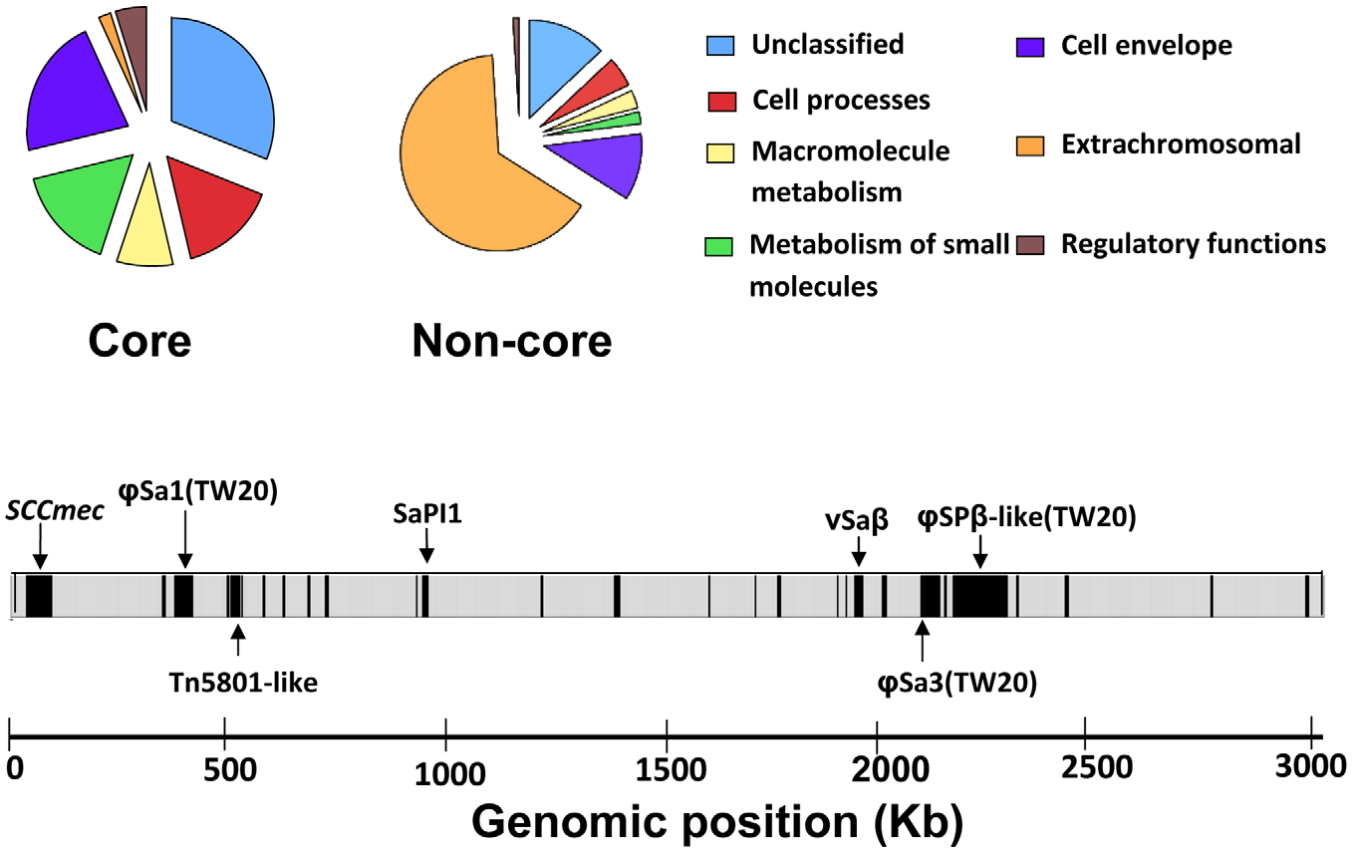
Functional classification of genes assigned to core and non-core regions based on an adapted version of Riley's classification. The non-core is dominated by “extrachromosomal elements”. The positions of non-core along the genome are shown in black, with well characterised mobile elements highlighted (*SCCmec* = resistance island (plus SCC*mer*); ϕSa1(TW20), ϕSa3(TW20), ϕSPβ-like(TW20) = prophage; Tn*5801*-like = transposon; SaPI1 = pathogenicity island; νSaβ = genomic island).

Based on the core definition of Harris et al [16] (described in Methods), and including intergenic SNPs, we note a total of 4250 core SNPs (affecting 1492 core genes), and 2459 non-core SNPs (affecting 257 non-core genes) within 63 isolates composing the ST239 dataset. In total, there are 2562750 bp within the core region and 454915 bp within the non-core region, hence the overall SNP densities are 1.658×10^−3^ SNPs per site for the core, and 5.405×10^−3^ SNPs per site for the non-core. The higher SNP density in the non-core reflects the high proportion of mobile (“extra-chromosomal”) elements, including prophage, genomic islands and other MGEs (Figure 1) [14]. Note that the “extrachromosal” category in Figure 1 refers to the likely horizontal origin of these genes, and all SNPs are physically located on the chromosome. Short IS elements may also be assigned as non-core, as will regions that have encountered small deletions (Figure 1).

In order to check that the data is not overly biased by the presence of a large number of non-core genes in a small number of isolates, we checked how many non-core genes were present in only one strain, any two strains, three strains and so on. As many non-core genes are partially mapped, we took both the most inclusive definition of gene “presence” (at least one mapped read within the CDS), and the most exclusive definition (100% of the CDS is mapped) (supplementary Table S1). Plotting the cumulative proportions of non-core genes present in 1, 2 …63 isolates based on these two definitions revealed that essentially all non-core genes were present in at least 13 isolates, and >50% of non-core genes were present in at least 40/63 isolates (supplementary Figure S1).

**Figure 2:**
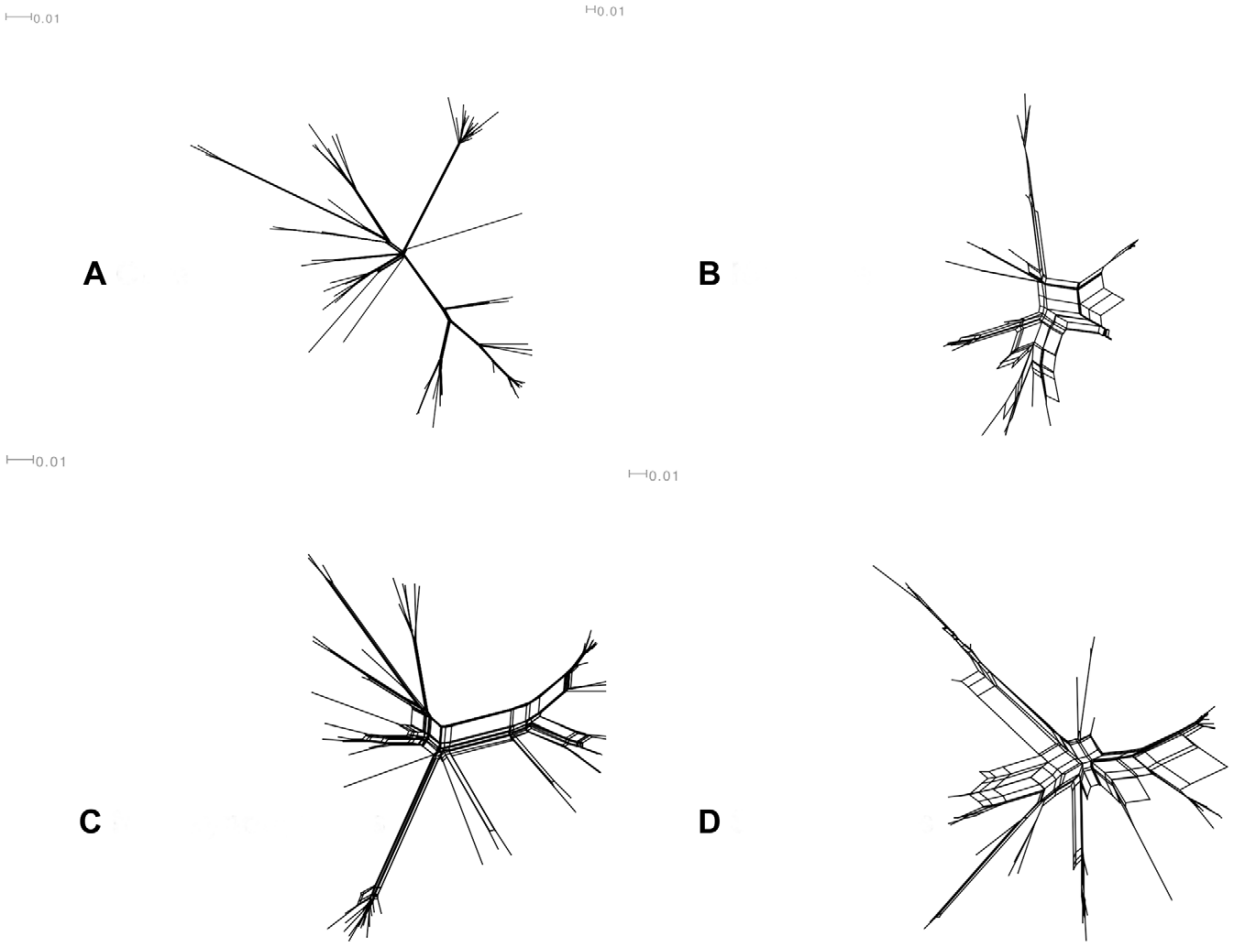
Neighbour-net networks constructed using Splits Tree 4.0. Four subsets of the SNP data were used: A – synonymous and non-synonymous SNPs in the core, B – synonymous and non-synonymous SNPs present in the non-core, C – all non-synonymous SNPs, and D – all synonymous SNPs. Taxon labels were removed for the sake of clarity. Uncorrected p distances were used and the networks drawn using the equal angle method. The networks show extensive reticulation in B and D, but less in A and C. This is consistent with high rates of recombination in the non-core, relative to the core, and in synonymous SNPs as these are enriched in the non-core. The Phi test for detecting recombination, as implemented in Splits Tree, was also used on these subsets. This was highly significant for B and D (both p values<0.001) but not significant for A and C (both p values>0.1).

Harris et al noted that recombination has been rare in the core, a view supported by the paucity of homoplasies [16]. In contrast, the frequent horizontal transfer, mosaic structure and modular nature of mobile elements in the non-core is consistent with frequent recombination [43]. To directly compare levels of recombination in the core and non-core, we constructed neighbour-net networks, as implemented in Splitstree 4 [44], based on core and non-core SNPs separately (Figure 2, A and B). Whereas no reticulation is apparent in the core genome (Figure 2A), confirming low rates of recombination, extensive reticulation is noted for the non-core, consistent with high rates of recombination (Figure 2B). This impression is borne out by the phi test which provided significant evidence of recombination for the non-core (P<0.001), but no significance evidence for recombination for the core (P>0.1).

### Distribution of synonymous and non-synonymous SNPs

Table 1 gives the total numbers and percentages of synonymous, non-synonymous and intergenic SNPs in the core and non-core. Whereas there are more than twice as many non-synonymous than synonymous SNPs in the core genome, for the non-core the reverse is true. A chi-squared test confirms that the core is highly significantly enriched for non-synonymous changes, relative to the non-core (χ^2^ = 719.325, p<0.00001). Given higher rates of recombination in the non-core, this observation provides a simple explanation as to why there is more reticulation when networks are reconstructed from all synonymous SNPs as compared to all non-synonymous SNPs (Figure 2, C and D). The non-core therefore differs from the core in three respects: i) a higher SNP density, ii) a higher rate of recombination and iii) a higher proportion of synonymous change.

**Table 1:** Number of non-synonymous, synonymous, and intergenic SNPs for core and non-core regions in *S. aureus* ST239.

	Non-synonymous (%)	Synonymous (%)	Intergenic (%)
**Core**	2187 (51.5)	1016 (23.9)	1047 (24.6)
**Non-core**	687 (28)	1387 (56.4)	385 (15.6)
**Non-core clustered**	207 (21)	694 (70.4)	85 (8.5)
**Non-core dispersed**	476 (32.4)	695 (47.3)	298 (20.3)

We expanded this analysis by dividing the non-core SNPs into two approximately equal subsets; dispersed (<5 SNPs per 100 bp; n = 1469) and clustered (>5 SNPs per 100 bp; n = 986). We note over twice as many non-synonymous SNPs in the dispersed set (n = 476) compared to the clustered set (n = 207). In contrast, the numbers of synonymous SNPs are almost identical in the two subsets (694 and 695 respectively). This demonstrates that a higher proportion of synonymous SNPs are noted within regions of increased SNP density, even when only considering different non-core genes (χ^2^ = 72, p<0.005; Table 1; Supplementary Figure S2). We note that the non-core regions of high SNP density tend to correspond to the mobile elements (e.g. categorised as “extrachromosomal” in Figure 1). Non-core genes not included in this category are less SNP dense and show approximately equal numbers of non-synonymous (n = 148) and synonymous (n = 144) SNPs.

### Among site rate variation

To complement the analyses described above we used BEAST to examine the degree of rate variation for the core and non-core SNPs. This approach provides an additional means to examine the relative strengths of selection on the core and non-core genome. In the absence of strong selection we would not expect significant rate variation between synonymous and non-synonymous SNPs. In contrast, strong purifying selection would decrease the rate of change for non-synonymous SNPs, resulting in site variation. Model selection was carried out as described in Methods, and the Akaike Information Criterion (AIC) and Bayesian Information Criterion gave essentially identical results. The best DNA substitution scheme for the core data set was TVMef, whereas the non-core selected the TVM scheme (these schemes are identical except the former assumes equal base frequencies). Far greater rate variation among sites was found in the non-core data than in the core, which is expected as non-core SNPs represent imports from multiple donor lineages. None of the robustly supported models required correction for rate variation among sites in the core genes, whereas all the robustly supported models for the non-core data required a correction for rate variation among sites. Furthermore estimates of the alpha parameter were far lower for the non-core data (∼3.7) than for the core data (approaching 100).

**Figure 3:**
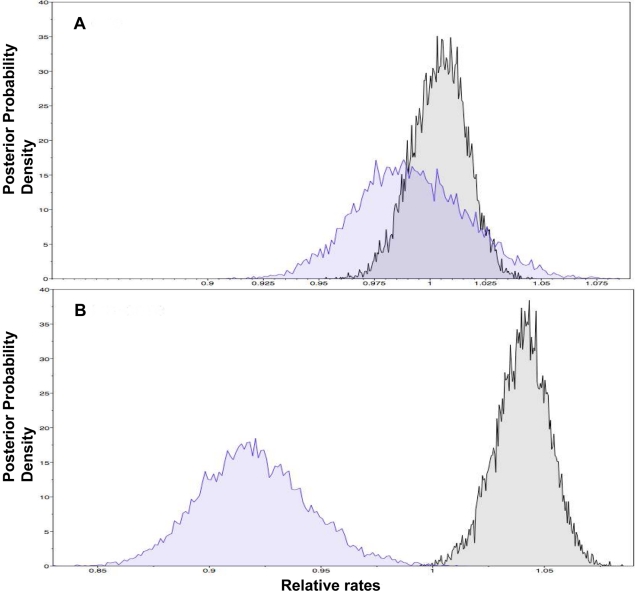
Posterior probability densities for the relative substitution rates. Synonymous and non-synonymous SNPs were considered for the core (A) and non-core (B). One partition contained only non-synonymous SNPs (blue), whereas the other contained synonymous SNPs (grey). In the core data set (A) the synonymous partition (mean = 1.0041; 95% CI 0.9797–1.0281) is not significantly different from the non-synonymous partition (mean = 0.9918; 95% CI 0.9438–1.0407), although the former is slightly higher. In contrast, for the non-core data set (B) the relative rate for synonymous partition (mean = 1.0401; 95% CI 1.0159–1.0635) is significantly higher than the non-synonymous partition (mean = 0.9199; 95% CI 0.8731–0.9681).

Whereas neither synonymous or non-synonymous SNPs (when considered separately) required correction for rate variation in the core data, both required this correction for the non-core, and in this case non-synonymous SNPs showed more rate variation (alpha parameter = 2.86) than synonymous SNPs (alpha parameter = 4.73). Figure 3A shows the relative rates of change for synonymous and non-synonymous SNPs in the core. Although the distributions substantially overlap, the mean rate is marginally faster for the synonymous SNPs and a larger variance is evident for the non-synonymous SNPs. This is consistent with a wider range of selective consequences of non-synonymous SNPs, and the initial purging of the most deleterious class. Figure 3B shows the relative rate distributions for the synonymous and non-synonymous SNPs in the non-core. In this case the distributions are significantly non-overlapping, consistent with the selective removal of a far higher proportion of slightly deleterious non-synonymous SNPs (the mean rate and confidence intervals are given in the figure legend).

### Time dependence versus effective population size

The above analyses demonstrate that core SNPs are the least densely clustered, and show a much lower proportion of synonymous change than the non-core. As core SNPs are likely to have emerged more recently (on average) than non-core SNPs, this is consistent with the time dependency of dN/dS noted previously between genomes of differing levels of divergence [34]. However, it is not clear whether this time dependency is sufficient to explain the difference between the core and non-core or whether other factors are playing a role. For example, it is possible that a reduction in the genome-wide effective population size may have resulted in increased drift on core genes. Low rates of recombination also decrease the local population size by increasing background selection, and this could contribute to the relative paucity of neutral variation in core genes.

**Figure 4:**
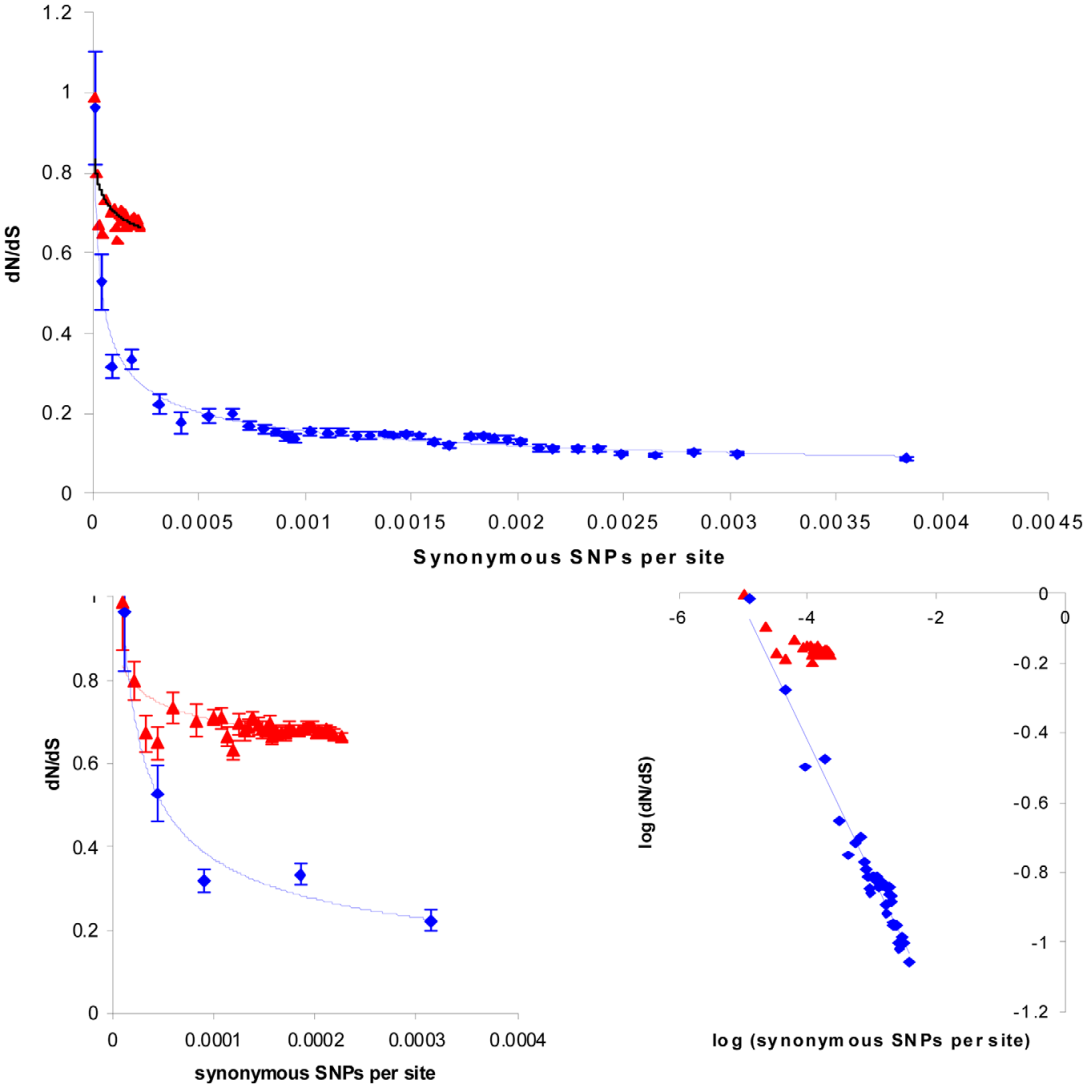
The time dependence of dN/dS in the core and non-core genome. We calculated this ratio over all 1953 pairwise strain comparisons separately for the two sets of genes. We also calculated, for each pairwise comparison, the number of synonymous SNPs per synonymous site as a measure of divergence. We divided the results into bins of 50 pairwise comparisons according to increasing divergence (i.e. the first bin corresponding to the 50 least diverged comparisons). For each bin we plotted the average dN/dS against the average divergence, with standard errors calculated form all 50 data points (main panel). The data for the core is shown in red, the non-core in blue. To clarify the plot for the core data, which is far less diverged than the non-core, we re-scaled the figure (bottom left) to include only the five most conserved non-core bins. The trajectory for the non-core data fits closely to a power law (R^2^ = 0.96) as shown by the linear relationship when both axes are log-transformed (bottom right).

In order to disentangle these different effects we first controlled for time dependence by comparing core and non-core genes at similar levels of divergence. We calculated dN/dS for all 1953 pairwise comparisons of the 63 isolates, separately for the core and the non-core regions. For each pair we estimated divergence time by calculating the divergence at synonymous sites (again for the core and non-core separately). Figure 4, main panel, plots the average synonymous site divergence against the average dN/dS for 39 bins, each of 50 pairwise comparisons. This plot confirms the high dN/dS ratio in the core, relative to the non-core, and reveals that the maximum synonymous SNP density in the core is over an order of magnitude lower than that of the non-core. Figure 4, bottom left, rescales the plot in order to clarify the patterns in the core data and the most conserved 5 bins of the non-core, where levels of divergence overlap. We note that the most conserved bins for both the core and non-core correspond to a dN/dS ratio approaching parity, thus the most recent mutations have been subject to very little purifying selection, regardless of whether they emerged in core or non-core genes. However, whereas the subsequent decrease in dN/dS for the non-core is striking, and closely fits a power law (R^2^ = 0.96) (Figure 4, bottom right), the plot for core genome follows a very shallow trajectory. This strongly suggests that time dependence alone cannot explain the differences in dN/dS between the core and non-core genomes. A decrease in the efficiency of purifying selection owing to a reduction of the effective population size might explain the relatively slow purging of non-synonymous mutations in the core [34]. This weakened selection may have resulted from the rapid global spread of ST239, combined with specialised adaptation to the hospital environment. Such an ecological shift would disproportionately affect the core SNPs as these are more likely to have emerged *de novo* since the emergence of the clone. The high rates of recombination experienced by non-core genes might also act to buffer against increased drift acting elsewhere on the genome by maintaining a higher local effective population size.

### Recombination is not linked to inflated neutral diversity over extraclonal comparisons

It is well documented that in eukaryotic genomes low rates of recombination are associated with low levels of neutral variation [45], [46], [47], [48], [49], and that recombination facilitates protein adaptation [50]. An important mechanism underlying this is background selection, whereby the selective purging of deleterious mutations results in the loss of neutral variation at linked sites [42]. The emergence of an adaptive mutation has a similar effect through hitch hiking [41], and in both cases the size of the genomic region affected is determined by the local rate of recombination [40]. The effect will be stronger in lowly recombining regions of the genome where more neutral variation remains linked to the site under selection. The role of positive selection on core genes should also be considered [29], along with the possibility that recombination is mutagenic [51] (although it is unclear in this latter case how this can explain the strong enrichment of synonymous change in recombining genes).

When considering these alternative hypotheses, it is important to emphasize that the analyses described thus far considers isolates within a single clone, which probably emerged under 50 years ago [16] and thus corresponds to a tiny fraction of the diversity in the species as a whole. Our hypothesis of time dependence is broadly distinct from the alternative explanations listed above, in that it predicts that the high proportion of non-synonymous change in core genes should be substantially moderated over greater levels of divergence, such that the differences in dN/dS between core and non-core genes should diminish. Comparing a more representative sample of *S. aureus* genomes therefore provides a simple means to test our model of time dependence against these alternative hypotheses. To this end, we calculated the dN/dS ratio between core orthologs in TW20 (see Methods) and three other *S. aureus* genomes representing a range of divergence times: i) within the same clonal complex (TW20 vs USA300), ii) within different clonal complexes (TW20 vs MRSA252), and iii) highly divergent (TW20 vs MSHR1132). This latter genome corresponds to the unusually diverged *S. aureus* genotype ST75 which has been recorded in Northern Australia [52], Cambodia [53] and French Guiana [54].

**Figure 5:**
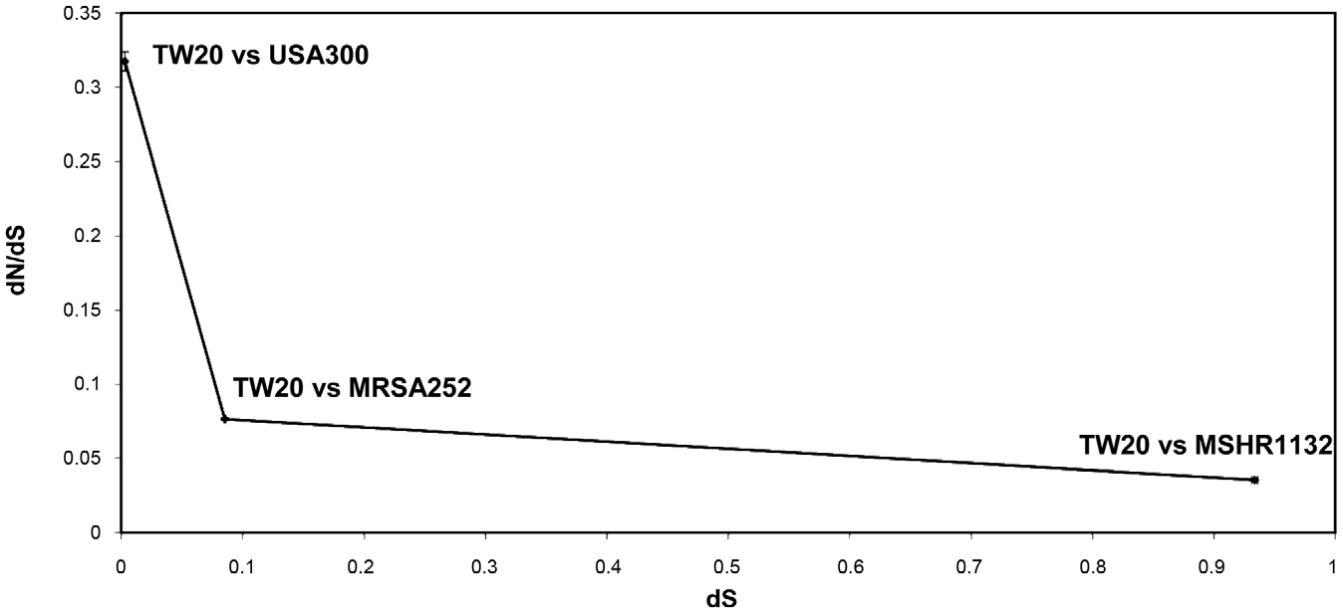
The mean dN/dS of core orthologues plotted against dS. Three genome sequences were compared in a pairwise fashion to the TW20 (ST239) reference. These genomes represented different levels of divergence, and the analysis confirms that the dN/dS within core genes decreases over time. Error bars represent the standard error from the re-sampled data (see Methods).

For each of the three pairwise comparisons we calculated the mean dS and dN/dS, along with a standard error calculated by re-sampling the data as described in Methods. Note that we omitted those core genes corresponding to the large replacement region as described below. Figure 5 confirms that the dN/dS between core genes decreases with increasing synonymous site divergence. The mean dN/dS ratio for the intermediate comparison (TW20 vs MRSA252), which provides the most representative comparison for the species as whole, is below 0.1. We note this is lower than the average dN/dS observed for the non-core regions within the ST239 clone (0.18). This suggests that the difference between core and non-core genes has not only been diminished, but has reversed, in that non-core genes show a higher dN/dS than core genes when greater divergence times are considered. To confirm this we also calculated dN/dS separately for 57 orthologous non-core genes, identified on the basis of reciprocal Fasta best hits (as described previously [14]), between the most divergent comparison (TW20 vs MSHR1132) (Supplementary Table S2). The average dN/dS for these non-core genes is ∼0.1, which is over double the average of ∼0.04 noted in the core genes for the same comparison. Thus the relative inflation of neutral variation associated with non-core genes within the ST239 clone is only apparent over very short phylogenetic distances, and this argues against a strong role for background selection, positive selection on core genes or the mutagenic effects of recombination as alternative explanations.

### Patterns of dN/dS within the large homologous replacement of ST239

The unique hybrid structure of the TW20 genome presents an opportunity to test whether the differences we observe between the core and non-core genes result from differing selection pressures on these two sets of genes. This large (635 kb) homologous replacement in the ST239 genome has affected many core and non-core genes, but for this analysis we only consider core genes where orthologs can be robustly identified in both parental genomes (see Methods). We compared patterns of dS and dN/dS within and outside the replacement region using the full genome sequences corresponding to the recipient clone (USA300; ST8) [55], the donor clone (MRSA252; ST36) [23] and the resultant hybrid (TW20; ST239) [14]. In order to gauge significance, we used a re-sampling procedure as described in Methods.

**Figure 6:**
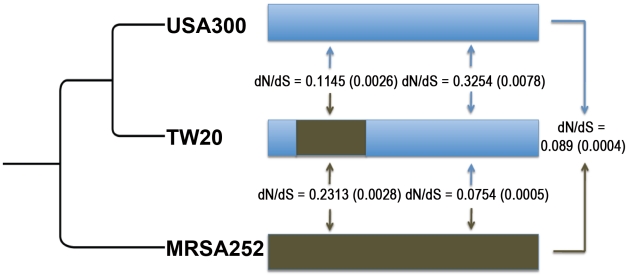
Mean dN/dS values are shown (with standard errors in parentheses) between *S. aureus* ST239 (TW20) and *S. aureus* USA300 (USA300), and between TW20 and *S. aureus* MRSA252 (MRSA252). The brown section in TW20 depicts the large (635 kb) recombination event which originated from a close relative of MRSA252, the full genome is ∼3 Mb (not drawn to scale). On the left, the dendrogram illustrates the relationships between these strains.

Overall patterns of divergence (dS) confirm that the replacement region within TW20 (hybrid) is much more similar to MRSA252 (the donor) than USA300 (the recipient), whilst the reverse is true for the rest of the genome (Supplementary Figure S3). When TW20 and USA300 are compared, the dN/dS ratios are much lower within the replacement (∼0.12) than the rest of the genome (∼0.33) (Figure 6). This is entirely expected, as the replacement represents an import from a diverged lineage so, similar to the non-core, should be relatively enriched for synonymous changes. The link between inflated neutral diversity and recombination therefore holds similarly for core genes which have been replaced by diverged homologous imports as it does for non core genes, and is therefore unlikely to be related to gene specific selection pressures. As a further check we confirmed that when the TW20 (hybrid) and MRSA252 (donor) genomes are compared the reverse is true; the dN/dS ratio within the replacement is higher (0.23) than the rest of the genome (0.08) (Figure 6).

### Evidence from *Clostridium difficile*

The analysis above demonstrates that diverged homologous replacements may result in a relative local enrichment of synonymous change. In order to check whether a similar pattern could be observed in other species, we used the genome-wide SNP data for 25 isolates belonging to a single hypervirulent clone of *Clostridium difficile* presented by He et al [18]. These data are well suited to this analysis, as two of these isolates (bi11 and bi4) exhibit regions of high SNP density consistent with large-scale homologous recombination from outside of the clone. Following He et al. [18] we assign these blocks of high density SNPs as having arisen by homologous recombination. These blocks of recombination account for the vast majority (89.4%) of all the SNPs detected within all the 25 isolates of this clone. Whereas the average SNP density across all strains outside of these blocks was only 6×10^−5^ SNPs per site, all the blocks correspond to a SNP density at least an order of magnitude higher than this, and the average SNP density within the blocks was 1.4×10^−3^ SNPs per site. Because the recombination blocks within strains bi11 and bi4 correspond to such striking peaks of SNP density, we simply identified all the 1553 SNPs within these two strains likely to have arisen by recombination by visual inspection of the SNP alignment (Supplementary Figure S4). We then compared the number of synonymous, non-synonymous and intergenic SNPs within the blocks with the remaining 184 SNPs (Table 2). Consistent with expectation, a significant enrichment of synonymous SNPs was observed for those changes corresponding to the regions of densely clustered SNPs, and therefore assigned as having arisen by recombination (χ^2^ = 34, p<0.005).

**Table 2:** Number of non-synonymous, synonymous, and intergenic SNPs for recombined and non-recombined regions in a hypevirulent clone of *C. difficile*.

	Non-synonymous (%)	Synonymous (%)	Intergenic (%)
**Non-recombined**	119 (64.7)	30 (16.3)	35 (19)
**Recombined**	737 (47.5)	609 (39.2)	207 (13.3)

## Discussion

Here we have exploited four complete *S. aureus* genome sequences, and revisited the genome-wide SNP datasets of *S. aureus* [16], and *Clostridium difficile* [18] to examine how recombination impacts on the level of neutral variation within recently emerged bacterial clones. A key starting point is the high level of non-synonymous change among very recently emerged mutations, and a commensurate decrease in dN/dS over divergence time [18], [34], [37], [38], [56], [57], [58], [59]. For *S. aureus*, which is a highly structured (clonal) population, this is evident as a preponderance of non-synonymous change within clones (dN/dS∼0.7) compared to between clones (dN/dS∼0.1) [23], [24]. Given this framework, it is clear that the importation of DNA into a bacterial clone from elsewhere is predicted to introduce a relative preponderance of synonymous change, and this is strongly supported by our data. Furthermore, we note that the proportion of synonymous change increases as the donor lineage is more divergent. This confirms that the relative enrichment of synonymous SNPs over time equally applies to genes acquired from disparate lineages within a single genome as it does to overall levels of divergence between pairs of genomes. The relationship of dN/dS to synonymous site divergence in the non-core fits closely to a power law (R^2^ = 0.96). Although more work is required to elucidate the properties of these plots for different datasets, we note that the decrease in dN/dS over greater scales of divergence (between clones; Figure 5) appears to show a broadly similar relationship.

Whilst it is clearly necessary to account for the time-dependence of dN/dS when comparing the strength of selection between genes, genomes or populations over very short time-scales, this model does not fully explain the difference we observe between the core and non-core genes in *S. aureus*. Even when controlling for this effect (i.e. comparing genes at the same level of divergence) the core genes show an enrichment of non-synonymous SNPs relative to the non-core. Rocha et al showed that differences in the trajectory of the decrease in dN/dS over time can be explained by changes in the effective population size [34]. This effect has been examined in detail by comparing trajectories of dN/dS over time in *E. coli* and highly specialised *Shigella* clones [36], and the impact of increased drift through bottlenecking has also been discussed in *Mycobacterium bovis* [60].

It is possible that the epidemiological characteristics of ST239 may reduce the effective population size, thus weakening the efficiency of purifying selection. Since its emergence in the mid 1960s this clone has disseminated globally, and it has been estimated that it currently causes >90% of all cases of hospital-acquired MRSA within regions which together account for >60% of the global population [7]. In contrast to methicillin sensitive *S. aureus* (MSSA), which are typically carried asymptomatically in the community [61], ST239 is very rarely noted outside of the hospital environment. Direct transmission between hospitals must therefore play a large role in the global dissemination of this clone, and this mode of dissemination will incur substantial bottlenecking if very limited variation is introduced into any given hospital. Commenting on the large number of impressively reinforced, yet extinct, species in the fossil record, Haldane remarked that “… in some cases the species literally sank under the weight of their own armaments” [62]. In the case of *S. aureus* ST239, which has already been replaced in Western Europe and is currently being replaced in other parts of the world [63], [64], the “armament” of multiple drug resistance may be costly in terms of cell function and resources, but also in terms of restricting the competitiveness of the clone to those health care settings where antibiotics are most aggressively deployed.

The argument above has two important implications. First, it raises the possibility that rapidly emerging clinically important clones which are exclusively maintained in health-care settings are inevitably self-limiting. This may help to account for the cycles of clonal expansion and replacement commonly noted within *S. aureus* and other pathogen populations [65]. It will be interesting to examine this further by comparing the trajectories of dN/dS over time in samples representing different ecological constraints and effective population sizes. Second, this analysis provides a novel approach for detecting increased drift through comparisons between core and non-core genes within a single genome. This contrasts with whole genome comparisons of different lineages as carried out previously for *E. coli* and *Shigella* [36]. The current study also illustrates the importance, and potential, of considering evolutionary processes within the context of the age and history of different genomic regions, rather than purely in terms of direct selective effects. Such a perspective underpins studies on the likely fate of acquired genes [66], [67], [68] or the effects of age on the functional and selective stability of proteins [69].

To what extent can selection at linked sites, leading to variation in local effective population size (as determined by the rate of recombination), explain our data? Although background selection [42] has been discussed extensively for eukaryotes, its role in bacterial evolution remains almost completely unknown. Touchon et al demonstrated a lower rate of recombination, a lower value of Tajima's D and a higher dN/dS ratio around the terminus of replication in *E. coli* [67]. These authors interpreted the co-occurrence of low rates of recombination and an enrichment of slightly deleterious change as evidence of background selection. Whereas we have argued that recombination introduces neutral variation into the genome from a diverged lineage, an advocate of background selection would argue that recombination has saved much of the neutral variation that has emerged *de novo* within the genome which would otherwise have been lost. As illustrated by Figure 4, main panel, this would need to be a very powerful force because the maximum level of neutral variation in the non-core of the ST239 data is at least an order of magnitude greater than that in the core.

We note in this context a broad distinction between recombination in eukaryotes, where the evolutionary consequences tend to be viewed in terms of the process itself (e.g. background selection, biased gene conversion or the Hill-Robertson effect), and recombination in bacteria, which (because it is less common, but can occur over large phylogenetic distances) is typically more simply viewed in terms of how the incoming SNPs (or genes) directly impact on the genome. We argue that our primary observation –the relative enrichment of synonymous SNPs within recombined regions at *intra*-clonal (but not *inter*-clonal) scales– is most parsimoniously explained by this latter perspective. Although the efficiency of purifying selection may have been compromised within the ST239 clone, our analysis confirms the decrease of dN/dS on the core genome over longer evolutionary time-scales which encompass the variation within the species. This means that the difference in the proportion of neutral variation between the non-recombining core and the recombining non-core disappears, or is even reversed, after the very initial (intra-clonal) stages of diversification. It is not obvious how to reconcile this observation with background selection, although we do concede the possibility that expansion of the “population” in this way may have unpredictable consequences concerning the relative effective population sizes of highly and lowly recombining regions. If positive selection played a major role in the emergence of high levels of non-synonymous changes in the core, or if the putative mutagenic effect of recombination enhanced neutral variation in the non-core, then we would expect these patterns to be maintained over greater levels of divergence.

The analysis of the large homologous replacement in TW20 suggests that it is not the process of recombination *per se* which acts to inflate neutral variation, as this effect is apparent only in those cases where the imported region (MRSA252-like) is more divergent than the two comparator genomes (USA300 and TW20) are to each other. If this is not the case, then recombination will have the opposite effect and remove neutral variation that has accumulated between the parental lineages (this applies when comparing TW20 with MSSA252). Finally, as this analysis is based on the core genes, the differences we observe in the SNP data are unlikely to be due to differing selective constraints acting on core and non-core regions.

One curious observation remains. For both the *S. aureus* and *C. difficile* data, the percentage of intergenic SNPs is lower when the changes are assigned as having been acquired horizontally from outside the clone (non-core or recombined; Tables 1 and 2). In the case of the non-core *S. aureus* data, this might be partly explained by difficulties in mapping very short and diverged intergenic sites in phage. However, this cannot explain the *C. difficile* data, where we note there is no significant difference between the numbers of intergenic and non-syonymous SNPs in the recombined and non-recombined datasets (in contrast, the difference between intergenic and synonymous SNPs is highly significant). Assuming that intergenic and synonymous SNPs are approximate selective equivalents, at least compared to non-synonymous SNPs, then this is the opposite trend to that expected. The reasons for the relative paucity of intergenic (compared to synonymous) SNPs within the non-core (*S. aureus*) or recombined (*C. difficile*) datasets may hint at selection on intergenic sites, and this observation clearly warrants further attention.

In conclusion, here we demonstrate that the dN/dS ratio varies according to the level of divergence and the past history of recombination between different genes, as well as due to population level effects associated with ecological specialisation. Whilst further work is required to elucidate the possible effects of selection at linked sites in natural bacterial populations, it is clear that variation imported into recently emerged bacterial clones from diverged lineages will contain a relatively high proportion of synonymous SNPs. This effect is not restricted to non-core genes, and the analysis on the *C. difficile* data demonstrates it extends to species other than *S. aureus*. As imported SNPs have been pre-filtered by purifying selection, so one might expect to see a general increase in the relative rate of recombination moving backwards in the tree as *de novo* mutations will tend to be purged more rapidly than recombination events. Recent studies have discussed the rapid rate of mutation at the very tips of the trees, which decreases over time as mutations are purged [35], [70], but it is not yet clear whether the rate of recombination shows a similar decrease. More practically, we propose that the enrichment of local synonymous change within bacterial clones might be used as an additional diagnostic to identify recombination events, thus facilitating detailed studies into their size and frequency or, through their subsequent removal, more robust phylogenetic analysis. Finally, although this work has focussed on the selective removal of slightly deleterious non-synonymous changes, future studies might also consider possible selective costs of SNPs at synonymous and intergenic sites [36], [71], [72], [73].

## Materials and Methods

### Definition of core and non-core

We used the core and non-core SNP datasets as previously defined by Harris et al [16]. The core genome was identified conservatively and objectively, as all sequences >1 kb that are present in all 63 isolates. Note that non-core regions present in the query strains, but absent in the reference sequence (TW20), are excluded. Rather than representing novel elements, the non-core SNP data in this study corresponds to the allelic variation of the accessory elements present in TW20 (e.g. polymorphisms present between closely related variants of the same non-core element). We computed the proportion of synonymous, non-synonymous, and intergenic SNPs for both core and non-core data sets using scripts written in Python.

### Neighbour-nets

We used four alignments in these analyses: i) all the synonymous and non-synonymous SNPs located in the core regions, ii) all the synonymous and non-synonymous present in the non-core regions, iii) all the synonymous SNPs irrespective of their presence in core or non-core regions, iv) all the non-synonymous SNPs regardless of their presence in core or non-core regions. For each alignment neighbour-nets were constructed using uncorrected p distances and were drawn using the Equal Angle method. Additionally, the Phi test for detecting recombination was conducted on all 4 alignments. All these analyses were carried out using SplitsTree 4 [44].

### Model selection

Statistical selection of models of nucleotide substitution was carried out using jModelTest [74] on an alignment of all SNPs for each data set, and on separate partitions (see below). Likelihood scores were computed for the different models. Corrections for unequal base frequencies and rate variation among sites were allowed, but we did not consider the correction for invariable sites as all SNPs are, by definition, variable. The Akaike Information Criterion (AIC) and Bayesian Information Criterion were used to perform model selection. The models used were: [TVMef SYM TVM GTR TVMef+G SYM+G TVM+G GTR+G TPM1 TIM1ef TPM1uf TIM1 K80 TrNef TPM1+G HKY TIM1ef+G TrN TPM1uf+G TIM1+G K80+G TrNef+G HKY+G TrN+G F81 JC F81+G JC+G].

### Bayesian analyses

We used the Bayesian methods implemented in BEAST [37] to estimate the rate of evolution for core and non-core SNPs. As the date of isolation was known for each single strain, we could calibrate the inferred phylogenies. In order to estimate relative rates, two data partitions (one consisting of the synonymous SNPs and other made up of non-synonymous SNPs) were used for the core and non-core data. These partitions were set up by editing the BEAST XML input files, as described in the BEAST manual (beast-mcmc.googlecode.com/files/BEAST14_Manual_6July2007.pdf). Since biological data sets will best fit a relaxed molecular model, which assumes independent rates on different branches, rather than a strict clock model, we used such an approach. The uncorrelated log-normal relaxed clock model was employed, using the GTR model with a gamma distribution for rate variation among sites. Substitution rate, rate heterogeneity, and base frequencies parameters were unlinked across partitions, otherwise default priors were used. For each analysis, one chain was run for 20,000,000 steps, and samples were taken every 2,000 steps. The first 2,000 steps were discarded as burn-in, and convergence was evaluated through TRACER, by examining the effective sample size (ESS) of the mean substitution rates and by examining trace plots of the likelihood scores.

### Analysis of the large homologous replacement

The genomes used were those of *S. aureus* TW20 (hybrid), *S. aureus* MRSA252 (donor), and *S. aureus* USA300 (recipient). We identified 2207 orthologues present in all three genomes by a reciprocal Fasta analysis performed previously [14]. Of these, 36 were discarded as pseudogenes leaving 2171 genes. The co-ordinates of the large homologous replacement (with respect to the TW20 genome) were taken as position 1… 427725 and position 2848037 … 3043210 (note there is only one contiguous block, but this passes through the origin of replication so is located at either end of the linear sequence). All orthologous genes which fell completely within these boundaries were assigned as REC (357 genes), whilst those orthologues which fell outside were assigned as NON-REC (1755 genes). Note that many of the genes falling within these boundaries in TW20 belong to non-core elements (e.g. SCC*mec*, SCC*mer*, prophage φSa1, Tn*552* and ICE*6013*), and as orthologues of many of these CDSs could not be identified in both MRSA252 and USA300 they were excluded. The exclusion of these non-core genes also allows us to test for gene specific effects between core and non-core genes. Individual alignments were made for each of the remaining genes as follows: first, using the protein sequences which are the translations of the genes we created protein alignments through MUSCLE [75] and, then, we used the program TRANALING, from The European Molecular Biology Open Software Suite [76], to generate alignments of the nucleic coding regions from the protein alignments (this was done in order to have DNA alignments in frame). To gauge the significance of differences in dS and dN/dS between the recombinant region and the rest of the genome, and to control for the different sizes of the datasets, we used a re-sampling procedure. We randomly sampled (with replacement) gene alignments until their concatenated length exceeded 378747 bp (the concatenated length of the 372 core genes of the recombinant region). This was repeated 200 times for the recombinant region and 200 times for the rest of the genome. For each replicate, pairwise dS and dN/dS values were computed from the concatenated alignments using the codeml program in PAML [77] to estimate synonymous substitution rates (dS), non-synonymous substitution rates (dN), and the ratio of the two (dN/dS). We specified “runmode = −2” in the control file to set pairwise calculations.

### dN/dS between core orthologues in TW20 and three other *S. aureus* genomes

In addition to the 3 genomes used for the analysis of the large replacement, we included the genome of *S. aureus* MSHR1132 that is distantly related to the other 3 genomes. In this analysis we kept only those orthologues located in the non-recombinant region (NON-REC orthologues, n = 1755 genes, see above). Using these orthologues, we generated 200 replicates of concatenated alignments following the re-sampling procedure mentioned above. Pairwise dS and dN/dS values were then computed for each replicate through PAML as previously described.

## Supporting Information

Figure S1 The cumulative proportion of non-core genes present in 1, 2 … 62 isolates. Genes were scored as present in a given isolate using two definitions: i) >0% of the CDS was mapped (blue line), or ii) 100% of the CDS was mapped (red line). The total number of non-core genes (present in <63 isolates) using the former definition is 418, whereas the total number of non-core genes using the latter definition is 632.
(DOC)

Figure S2 This figure illustrates that core SNPs are least dense and show a lower proportion of synonymous change than non-core SNPs. Furthermore, clustered non-core SNPs show a greater enrichment of synonymous change than dispersed non-core SNPs. The bar charts show the proportions of synonymous (red) and non-synonymous (blue) SNPs in the core, dispersed non-core, and clustered non-core, as computed from the data.
(DOC)

Figure S3 dS pairwise comparisons of the recombinant (rec) and non-recombinant regions (non-rec). The rec and non-rec dS values are shown for the comparison involving *S. aureus* ST239 (TW20) and *S. aureus* USA300 (USA) and for the comparison of TW20 and *S. aureus* MRSA252 (MRSA). The two boxes on the left show the recombinant region, whereas the two boxes on the right show the non-recombinant region.
(DOC)

Figure S4 Alignment of SNPs within the 25 hypervirulent strains of *C. difficile* described by He et al [18]. The number of each SNP is given in vertical format above the alignment (this does not provide positional information as only polymorphic sites are included). Bases identical to that observed in the reference are shown as a dot. The vast majority of SNPs correspond to large blocks of homologous recombination within two strains; bi4 and bi11. SNPs which are likely to correspond to these recombination events were identified by visual inspection and shown in bold, and the numbers of synonymous, non-synonymous and intergenic SNPs compared for the recombined and non-recombined SNP sets.
(DOC)

Table S1 The percentage of the CDS which is mapped for each gene in each isolate.
(TXT)

Table S2 dN/dS for 57 orthologous non-core genes in TW20 and MSHR1332.
(DOC)
