# Supplementary figures and images for: The Impact of Recombination on dN/dS within Recently Emerged Bacterial Clones
Source: PLoS Pathog. 2011 Jul 14;7(7):e1002129. doi: 10.1371/journal.ppat.1002129 (PMC3136474; doi:10.1371/journal.ppat.1002129)

Supplementary Figure S1


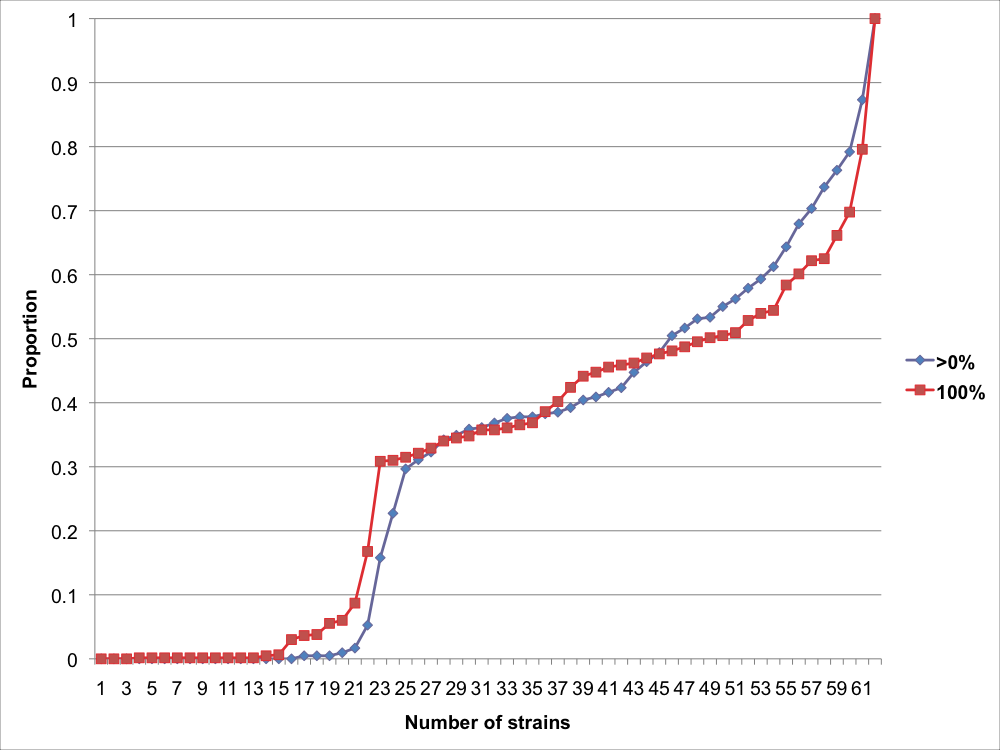

Supplement: Figure S1 — The cumulative proportion of non-core genes present in 1, 2 … 62 isolates. Genes were scored as present in a given isolate using two definitions: i) >0% of the CDS was mapped (blue line), or ii) 100% of the CDS was mapped (red line). The total number of non-core genes (present in <63 isolates) using the former definition is 418, whereas the total number of non-core genes using the latter definition is 632. (DOC) [file ppat.1002129.s001.doc]

Supplementary Figure S2


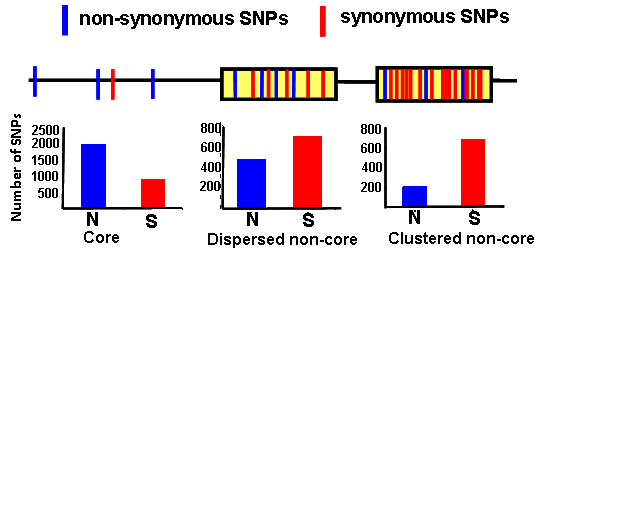

Supplement: Figure S2 — This figure illustrates that core SNPs are least dense and show a lower proportion of synonymous change than non-core SNPs. Furthermore, clustered non-core SNPs show a greater enrichment of synonymous change than dispersed non-core SNPs. The bar charts show the proportions of synonymous (red) and non-synonymous (blue) SNPs in the core, dispersed non-core, and clustered non-core, as computed from the data. (DOC) [file ppat.1002129.s002.doc]
