# Supplementary material for: The Impact of Recombination on dN/dS within Recently Emerged Bacterial Clones
Source: PLoS Pathog. 2011 Jul 14;7(7):e1002129. doi: 10.1371/journal.ppat.1002129 (PMC3136474; doi:10.1371/journal.ppat.1002129)
Supplement: Figure S3 — dS pairwise comparisons of the recombinant (rec) and non-recombinant regions (non-rec). The rec and non-rec dS values are shown for the comparison involving S. aureus ST239 (TW20) and S. aureus USA300 (USA) and for the comparison of TW20 and S. aureus MRSA252 (MRSA). The two boxes on the left show the recombinant region, whereas the two boxes on the right show the non-recombinant region. (DOC) [file ppat.1002129.s003.doc]

Supplementary Figure S3
