# Supplementary material for: The Impact of Recombination on dN/dS within Recently Emerged Bacterial Clones
Source: PLoS Pathog. 2011 Jul 14;7(7):e1002129. doi: 10.1371/journal.ppat.1002129 (PMC3136474; doi:10.1371/journal.ppat.1002129)
Supplement: Figure S4 — Alignment of SNPs within the 25 hypervirulent strains of C. difficile described by He et al [18]. The number of each SNP is given in vertical format above the alignment (this does not provide positional information as only polymorphic sites are included). Bases identical to that observed in the reference are shown as a dot. The vast majority of SNPs correspond to large blocks of homologous recombination within two strains; bi4 and bi11. SNPs which are likely to correspond to these recombination events were identified by visual inspection and shown in bold, and the numbers of synonymous, non-synonymous and intergenic SNPs compared for the recombined and non-recombined SNP sets. (DOC) [file ppat.1002129.s004.doc]

Supplementary Figure S4

11111111112222222222333333333344444444445555555555666666666677777777778]

[ 12345678901234567890123456789012345678901234567890123456789012345678901234567890]

Ref GCCGGTGCGACAAGCCTCGGCCCACTGAAACACCCTTGTCGTGGCTAGGGGCGCTCTCCCGTGTTTGCCCTGCGGGCGCC

2004013 .T..A......G.T..--....T..A...........................................A..........

2004102 .T......AG...T..--.......A.C...................................................T

2004118 .T..A......G.T..C-.......A...........................................A..........

2004163 .T....T....G.T..--.......A...........................................A..........

2006439 .T......AG...T..--.......-.C.........................................-.........T

2007140 .T.....AA....T..--.......-.................-.---.....................-.....T...T

2007218 .............T..--.......-...................---................................

2007825 .T.........G.T..--.T.....A....T...A.......-..........................-..........

2007833 AT.........-.T..--.......-.....-......-.-----------..-.-.---......--.-..-....-..

2007837 .T.........G.T..--.......A...........................................-..........

bi10 .T.A....A....T..--.......-................-......-.....-.......................T

bi11 .............TT.--.......A...G.............-.-..................................

bi13 .T......AG...T..--.......A.C................-........................-.........T

bi15 .TT.....AG...T.TCT......AA.C.........................................-.........T

bi2 .............T..--.......A................------.--..................-..........

bi3 .............T..--A..T.G.AA....G.........---.---..-....--............-C...A..T..

bi4 .....C....A..T..CTA.A....A.......T..**GAATTCTATGGTTATTA**.**GTCTTTACAGCCATA**.C...A...-.

bi5 .............T..--.......A......................................................

bi6 .T...........T..--.......A..G..............-----.......-..-...................T.

bi6p .T.........G.T..--.......A......T....................................A.A....T...

bi7 .T...........T..--.......A.........G.................T........................T.

cd855 .T......AG...T...........A.C...................................................T

cdsm .T.........GCT...........A......................................................

bi1 .............T..CT.......A..............................................TA......

[ 1111111111111111111111111111111111111111111111111111111111111]

[ 88888888899999999990000000000111111111122222222223333333333444444444455555555556]

[ 12345678901234567890123456789012345678901234567890123456789012345678901234567890]

Ref CGGCTACTCCGCGAGGCCTTGGGTCAATTGAGGCCATGAGAGTTGCGCCCACCTAAGGTACCGAACGATTTCCGCCGGTA

2004013 T...............................................................................

2004102 ................................A...............................................

2004118 T........G......................................................................

2004163 ................................................................................

2006439 ................................A...............................................

2007140 ..........-.....................A..............-................................

2007218 ....A...........................................................................

2007825 ..........-.....................................................................

2007833 TA.-----..-.-...-..-.....-.-.-.........-...-...---.....---..--..--.........-...-

2007837 T...............................................................................

bi10 ...-............................A...............................................

bi11 ...-.**GTCT**.**AGAGATTTCCAAACTGTGCTGA**.**TTG**.**AGAGACGATTTTTGATACCAACGTTACGAAGCCCTTATTCACG**

bi13 ..........-.....................A...............................................

bi15 ................................A...............................................

bi2 ....-.....-...............-.....................--..............................

bi3 ...A-...........................................-...............................

bi4 ..A......-......................................................................

bi5 ................................................................................

bi6 ..........-.....................A...............................................

bi6p ................................................................................

bi7 ................................A...............................................

cd855 ................................A...............................................

cdsm ....................................G...........................................

bi1 ................................................................................

[ 11111111111111111111111111111111111111122222222222222222222222222222222222222222]

[ 66666666677777777778888888888999999999900000000001111111111222222222233333333334]

[ 12345678901234567890123456789012345678901234567890123456789012345678901234567890]

Ref CAACGATCGATTGTCTGCCCGACCGAAACAAGCTCCAATTAAACGCTCAGCTATCATGAAAAGCCCCTCTGTCAGAACCA

2004013 ................................................................................

2004102 ...............................................................-..T.............

2004118 .............................................................................-..

2004163 ...............................................................-................

2006439 ..................................................................T.............

2007140 ...............................................................-................

2007218 .............................................................................-..

2007825 ................................................................................

2007833 --.-............-...........-........-.....----.--..-........---...------...--..

2007837 ................................................................................

bi10 .....-......................................-...........................-....-..

bi11 **TGGTAGCTAGCGTCACCTAAAGTTAGCGTGGATCTTGCACGGGTATCTGATCTCTGCAGGGGAATT**.**CTCAGTCAGTTAG**

bi13 ................-..............................................-..T..-.......-..

bi15 ................................................................................

bi2 ..-.........................-........-.-..--...........................-........

bi3 .....................................-.........................-................

bi4 .......................................................................G........

bi5 ................................................................................

bi6 .....................................-.........................-.............-..

bi6p ................................................................................

bi7 ................................................................................

cd855 ..................................................................T.............

cdsm ................................................................................

bi1 ................................................................................

[ 22222222222222222222222222222222222222222222222222222222222333333333333333333333]

[ 44444444455555555556666666666777777777788888888889999999999000000000011111111112]

[ 12345678901234567890123456789012345678901234567890123456789012345678901234567890]

Ref TGAAGGAGAAAGGTGAAACTAGGTCTACCCGCCAAAGCAAACCGTACATCTAACGGTTCACCTATGCTACCGCGCCGCGT

2004013 ...........................................................G.....C..C........A..

2004102 ..................................................................T...A.........

2004118 ...........................................................-.....C..C........A..

2004163 ...........................................................-.....C..C........A..

2006439 ..................................................................T...A.........

2007140 ...................-....--........................................T.............

2007218 ...................................--...........................................

2007825 .......................................................-...-.....C..C....T...A..

2007833 .------.--.---.--..-....---.....--.---..--.-......-........-.....C..C..---.--A..

2007837 ...........................................................G.....C..C........A..

bi10 ......-.-....--....-....--.-......................................T......-......

bi11 **CAGGAATAGGGACCAGGGTCGAACTCGTATATTGCGATGGGTAACGTGCTGGGAATC**.**A**.**TGCG**...............**C**

bi13 ..................................................................T...A.........

bi15 ..................................................................T..T......A...

bi2 ...-..-........-........--.-...........-.......--......-................-.......

bi3 .A......-..-.--.........--.............................................A......A.

bi4 .A.................................................................G...A..T...A.

bi5 ................................................................................

bi6 ........-......-....-...--........................................T........T....

bi6p ...........................................................-.....C..C...T....A..

bi7 ..................................................................T........T....

cd855 ..................................................................T...A.........

cdsm .........................................................G......AC..C........A..

bi1 ................................................................................

[ 33333333333333333333333333333333333333333333333333333333333333333333333333333334]

[ 22222222233333333334444444444555555555566666666667777777777888888888899999999990]

[ 12345678901234567890123456789012345678901234567890123456789012345678901234567890]

Ref TTGGAAACATCCAGCCCCCTGAATAACGTGTCAAGAGATGGGAGAGGCCACTGAAGTGGCGAGACGGCCAGCAAATTTTC

2004013 ....TC..-.GT......T..........A..................................................

2004102 .....C..-...............G....A.....C............................................

2004118 ....TC..-.GT...........C.G...A..................................................

2004163 ....TC..-.GT.................A..................................................

2006439 .....C..-...............G....A.....C............................................

2007140 .....C..-G...-A.........G....A..................................................

2007218 ........-......---...TT......A..................................................

2007825 ....TC..-.GT.-...............A......................................T...........

2007833 ....T-----GT.-.---..---.-....-..--.-...-..-.........--.....-...-.........-.-..--

2007837 ....TC..-.GT.................A..................................................

bi10 .....C..-.....A......--.G....A...........--.....................................

bi11 **CCAT**..G.-...T-...............A....C.**AGCAAAGAGA**.**TAGTCAGGACAATAGAGTAAT**.**GATTGG**.**CGCT**

bi13 .....C..-............-..G....A...-.C.....--.....................................

bi15 .....C..-...............G....A..................................................

bi2 .......--....-.-.............A...-..............................................

bi3 ........-.......AA.CA.......CA..GG........-................................C....

bi4 ........-......TAA.........A.A..GG..............................................

bi5 .......T-............--......A..................................................

bi6 ........-....................A...........--.....................................

bi6p ....TC..-.GT.A............T..A..................................................

bi7 ........-....................A..................................................

cd855 .....C..G...............G....AG....C............................................

cdsm ....TC.......................A.A..............T.................................

bi1 .............................A..................................................

[ 44444444444444444444444444444444444444444444444444444444444444444444444444444444]

[ 00000000011111111112222222222333333333344444444445555555555666666666677777777778]

[ 12345678901234567890123456789012345678901234567890123456789012345678901234567890]

Ref TGTCCTCTCTCATGCTTGTTTGGGTGCCAGTGTAGTAGAGCTAACTGTATTTATTAGTGTCTCGAGGTAGGGTGTCGAAT

2004013 ...................-...A........................................................

2004102 .......................A.......T.................................-..............

2004118 .......................A..................-.....................................

2004163 .......................A........................................................

2006439 -......................A.......T................................................

2007140 ...................-...A.-..................-.....................-.............

2007218 .......................A...-..............-.....................................

2007825 .......................A........................................................

2007833 -................---.-----.-...--.---..-.-----.---.-.....-----..---.............

2007837 .......................A........................................................

bi10 ...................-...--..-...............-.....................--.............

bi11 **CAATTCTCTCAGCATCCACACAA**-**CCGTCAG**..............-..................................

bi13 .......................A...-...T............--..................................

bi15 .......................A.......T................................................

bi2 .-.................-...A...-..............-..-.............-....................

bi3 ...................-...A........AGA.............................-...............

bi4 .......................A........A..**GCAGATCGGTCACTGCCGCCGCGAGTCAAGAACGAAACACTAGCC**

bi5 .......................A........................................................

bi6 .......................A-..-................--....................-.............

bi6p .......................A........................................................

bi7 .......................A........................................................

cd855 .......................A.......T................................................

cdsm .......................A........................................................

bi1 .......................A........................................................

[ 44444444444444444445555555555555555555555555555555555555555555555555555555555555]

[ 88888888899999999990000000000111111111122222222223333333333444444444455555555556]

[ 12345678901234567890123456789012345678901234567890123456789012345678901234567890]

Ref AGCTGATTTGACCATCGAAACGACACATGGCACTCTTATTGATAATGAGATCATCGTTGATGAGCGACTGATGCTTCAGA

2004013 .....-...-...........A....--...............................................--...

2004102 .....-....................--.................................-..................

2004118 .....-........-....G.A....--...............-................................-...

2004163 ..........................--T...............................-...............-...

2006439 .....-...-....-........-..--.......-......................................---...

2007140 .....-.-.-....-...........--..............................--.....-........---...

2007218 .....-....................--.......-.......................-.-..............-...

2007825 .....-........-.......G...--........-.......................................-...

2007833 ...---.-.-...--...--...-..--.......-------------...--.-.----------...-..--------

2007837 .....-...-...............A--................................................-...

bi10 .....-...-..-.-........-..--.-.....-.......--..........-..--.....-.......----...

bi11 .....-........-...........--...**GTCTGCGCCAGAGGCAGAG**.........---..................

bi13 ....--.-.-....-....-......--................................-................-..

bi15 .......-..................--....................................................

bi2 .....-.-.-.............-..--.......---......-..-..-.--.-...-....--...--.-------.

bi3 .....-.-.-..-......-......--.......---..............-......-....................

bi4 **GATCTCCC**.**AGTTGCTACC**....T..TA......................**ATCATACCAGGATATAGTCACCAACCTGAG**

bi5 .....-........-...........--..............................-..-..............-...

bi6 ....--.-.-....-........-..--.A..-..---..............-.-...-----..-...-...----...

bi6p ..........................--....................................................

bi7 .....-.-......-...........--........................-...........................

cd855 ................................................................................

cdsm ....................T...C.....T.................................................

bi1 ........C.................--....................................................

[ 55555555555555555555555555555555555555566666666666666666666666666666666666666666]

[ 66666666677777777778888888888999999999900000000001111111111222222222233333333334]

[ 12345678901234567890123456789012345678901234567890123456789012345678901234567890]

Ref TAGACAGTATCATGATAGCGGGGCTTCTGTTTATCGTTTCACGGAGAAGCCGCGCATCACCAAAGGTCATTCTGACAATC

2004013 ............-......A............................................................

2004102 ................................................................................

2004118 ...................A............................................................

2004163 ...................A............................................................

2006439 ................................................................................

2007140 ...........-....................................................................

2007218 ...........-....................................................................

2007825 ..................AA.....................................................-......

2007833 -...-..--.-----...-A-.---....-.--....-.....---..-----......---.........-.--.....

2007837 ...................A............................................................

bi10 ...........-......-...-.........-...............................................

bi11 ..........................T.....................................................

bi13 ................................................................................

bi15 ................................................................................

bi2 .........-..-..........--.......-..........................-..........-.........

bi3 ............-.......T..--.......................................................

bi4 **CGAGTGACGCTGCTGCGA**...**TATGC**.**GACCAGCTAGCCTCAAAGAGGTAT**.**TTTGCTTTTGGTAACTGCCTCAGTGGCT**

bi5 ................................................................................

bi6 ..........---..........--.-..............................................-......

bi6p ...................A...............................T............................

bi7 ................................................................................

cd855 ................................................................................

cdsm ...................A............................................................

bi1 ................................................................................

[ 66666666666666666666666666666666666666666666666666666666666777777777777777777777]

[ 44444444455555555556666666666777777777788888888889999999999000000000011111111112]

[ 12345678901234567890123456789012345678901234567890123456789012345678901234567890]

Ref ACTTTCCCCCTCGCCCTAAGTCCTGGTAATCGGCGTTCCGGTCGCTTCCGGTTGTGTATAAGGGTTCAGGAGTGCTATCC

2004013 ................................................................................

2004102 ................................................................................

2004118 ................................................................................

2004163 ................................................................................

2006439 ................................................................................

2007140 ........................................--......................................

2007218 ................................................................................

2007825 ..............................-.................................................

2007833 ..-.........-----....-..--------.-----.----..-.....-..--...--..........-........

2007837 .........................................-......................................

bi10 ..........................................-................-....................

bi11 ...............-.......................T........................................

bi13 .........................................--.....................................

bi15 ................................................................................

bi2 .............................-....-.....---........-............................

bi3 ........................................--......................................

bi4 **GTCCGTTTTTATATGAATGAATTCAACGGCTATTACCTT**.**ACTTTCCTTAACGAGACCCTGAAAACTCAAGAGATACATA**

bi5 ................................................................................

bi6 ............................-...........--......................................

bi6p ................................................................................

bi7 ................................................................................

cd855 ................................................................................

cdsm ................................................................................

bi1 ................................................................................

[ 77777777777777777777777777777777777777777777777777777777777777777777777777777778]

[ 22222222233333333334444444444555555555566666666667777777777888888888899999999990]

[ 12345678901234567890123456789012345678901234567890123456789012345678901234567890]

Ref CATGGTTTCACCGATCCGTCCATGTCGTTGGCCCTGCAGGAAAGACATGACCATACAGAGGCCCGTAGTGGTTTCGGCCT

2004013 ................................................................................

2004102 ................................................................................

2004118 ................................................................................

2004163 ................................................................................

2006439 ..................................................................-.............

2007140 ..................................................................-.............

2007218 ................................................................................

2007825 ..................................................................-.............

2007833 --................-..---.........................--.............-----..........-

2007837 ................................................................................

bi10 .................................................................-.-............

bi11 ................................................................................

bi13 ................................................................................

bi15 ................................................................................

bi2 ................................................................................

bi3 ................................................................................

bi4 **TGCCAACATGGTAGCTTAATTGCACTACCAATTTCAATTTGCGAGTCCAGATGCGTGAGAATTTACGCCAACCCTTATTA**

bi5 ................................................................................

bi6 ................................................................................

bi6p ................................................................................

bi7 ................................................................................

cd855 ................................................................................

cdsm ................................................................................

bi1 ................................................................................

[ 88888888888888888888888888888888888888888888888888888888888888888888888888888888]

[ 00000000011111111112222222222333333333344444444445555555555666666666677777777778]

[ 12345678901234567890123456789012345678901234567890123456789012345678901234567890]

Ref CGCCCCCCTTTGATACGTGTGCGTATGGGGGGTACAGACGAGTAAACGAGATAACGTGGATCATTATCACCTCATATGCG

2004013 ...................GT.........-.................................................

2004102 ..................A.............................................................

2004118 ...................GT...........................................................

2004163 ...................GT...........................................................

2006439 ..................A...........-.................................................

2007140 ..................A.-.........-.................................................

2007218 ..............................-.................................................

2007825 ...................GT.......-.-.................................................

2007833 -........-..-..----G--.-.--.-.-----......-..-.........-..--.........---.........

2007837 ...................GT.........-.................................................

bi10 ............-.....A......----.---.-.............................................

bi11 .............**CGTAC**.G.**TACGCATTAAACCTGAGTATAGGGGTAGAGCCGAACAAGCTGCCGCTGTACTCCGCATA**

bi13 ..................A...........-.................................................

bi15 ..................A.............................................................

bi2 ....................-...--.--.-.................................................

bi3 ..........................-.-.---...............................................

bi4 **TATTTTAACGCAG**...................................................................

bi5 ..............................-.................................................

bi6 .................-A....-....-.---...............................................

bi6p ...................GT...........................................................

bi7 ..................A.............................................................

cd855 ..................A.............................................................

cdsm ...................GT...........................................................

bi1 ................................................................................

[ 88888888888888888889999999999999999999999999999999999999999999999999999999999999]

[ 88888888899999999990000000000111111111122222222223333333333444444444455555555556]

[ 12345678901234567890123456789012345678901234567890123456789012345678901234567890]

Ref GAATTTTCGTCCTGTGAACCATTAGTCTCCCATCACTTGGTTCGCCTTGCCCAAGCACATCCACAACTACGTATCGCGAC

2004013 .........................................................................-...-..

2004102 ..............................................-.................................

2004118 .............................................-..................................

2004163 .............................................................................-..

2006439 .............................................-...............................-..

2007140 ...............................................-................G......-.-...-..

2007218 .........................................................................-...-..

2007825 ................................................................................

2007833 ..-...............-...-...--..-----...-......--.-......-..--.....---.-------.-..

2007837 ................................................................................

bi10 ................--...........................---.....................-....-.....

bi11 **ACGCCCATACTTCTCACTTAGCCGTCTCTTTGCTGTGCT**...............................-......-..

bi13 .....................................................................-...-...-..

bi15 .............................................................................-..

bi2 .............................................--......................---.-......

bi3 ..................-..................................................---.-...--.

bi4 .......................................**ACCTATAACATATGCATGTTCTAGT**.**GTCTTACGGTATTGT**

bi5 ................................................................................

bi6 ...............................-.............--.......................-..-...-..

bi6p ................................................................................

bi7 ................................................................................

cd855 ................................................................................

cdsm ................................................................................

bi1 ................................................................................

[ 11111111111111111111111111111111111111111]

[ 99999999999999999999999999999999999999900000000000000000000000000000000000000000]

[ 66666666677777777778888888888999999999900000000001111111111222222222233333333334]

[ 12345678901234567890123456789012345678901234567890123456789012345678901234567890]

Ref TTTCGACTTCATACGTATCTATCAGGTATCCGTGTCGCCTTATTGACCGGAGGGATGCTACGAGGATGGCATGATATATG

2004013 ................................................................................

2004102 ..................-.............................A...............................

2004118 ................................................................................

2004163 ................................................................................

2006439 ................---.............................A...............................

2007140 .................--.............................................................

2007218 .................-..............................................................

2007825 .................--.............................................................

2007833 ...........-...----..-.......----..--.........-.....--.--................--.....

2007837 .................--.............................................................

bi10 ................---......................................................-......

bi11 ..............-..-..............................................................

bi13 ..................-.............................A...............................

bi15 ................................................A...............................

bi2 ....-..........-.--.............................................................

bi3 .................-..............................................................

bi4 **CCCTAGTCCTGCGTACGCTCGCTGATCGCTATCACTATTCCGGCAGTA**.**AGATACCTACGTACACGCAATCCATCCCGCA**

bi5 .................-..............................................................

bi6 ................---.............................................................

bi6p ................................................................................

bi7 ................................................................................

cd855 ................................................A...............................

cdsm ................................................................................

bi1 ................................................................................

[ 11111111111111111111111111111111111111111111111111111111111111111111111111111111]

[ 00000000000000000000000000000000000000000000000000000000000111111111111111111111]

[ 44444444455555555556666666666777777777788888888889999999999000000000011111111112]

[ 12345678901234567890123456789012345678901234567890123456789012345678901234567890]

Ref CCTTCTTCCGATTCCCGTACATAGCTCCAACTGAACTGTACATATGAACTAGTCTATCTCGAGGTAAGAGTTGCTGCGCG

2004013 ................................................................................

2004102 ................................................................................

2004118 ................................................................................

2004163 ................................................................................

2006439 ................................................................-...............

2007140 .........................................-......................................

2007218 ................................................................................

2007825 ................................................................................

2007833 ...--....--------....--....-........--.--.--...-..--..----..-...-----.-.........

2007837 ................................................................................

bi10 ........................................................---...........-.........

bi11 ......................-.........................................................

bi13 ......................................................................-.........

bi15 ................................................................................

bi2 .....................-...................-...............-.......--.............

bi3 ..........................................................-.....................

bi4 **TAGCTGCTAACCATTTACCTCCGATCTTCCTGAGGTCTCGTGGGCACGTGGACTCGCTCTTGAACGGACTACATCAAATA**

bi5 ................................................................................

bi6 ....................................-....-...............-......................

bi6p ................................................................................

bi7 ................................................................................

cd855 ................................................................................

cdsm ................................................................................

bi1 ................................................................................

[ 11111111111111111111111111111111111111111111111111111111111111111111111111111111]

[ 11111111111111111111111111111111111111111111111111111111111111111111111111111112]

[ 22222222233333333334444444444555555555566666666667777777777888888888899999999990]

[ 12345678901234567890123456789012345678901234567890123456789012345678901234567890]

Ref TTTACCGTGTATGCATCCCCTAGGACCATGACGCGAGGCGCGTCGCGGGCATCGTAATCCAAGCCACGAGTCTGCTTACT

2004013 ........................G......................A...G...C........................

2004102 ................................................A..G............................

2004118 ........................G......................A...G...C........................

2004163 ........................G......................A...G...C........................

2006439 ................................................A..G............................

2007140 .............................................A..A..G............................

2007218 ...................................................G............................

2007825 ........................G......................A...G...C........................

2007833 ...-...--..--.-...---...--.....----.........---A...G...C.......................-

2007837 ........................G.....................AA...G...C........................

bi10 ...-.......-....................................A..G............................

bi11 ...................................................G............................

bi13 ................................................A..G............................

bi15 ................................................A..G............................

bi2 ........................-..........................G............................

bi3 .................................-.................G.TC.........................

bi4 **CCCCTTACACCCTTCCTTTTCGAA**.**TTGCAGTAAAGAATAATCTT**......G.....**GTTGGATAGTAGACAC**.**ACCGAC**

bi5 ...................................................G............................

bi6 ................................................A..GT...........................

bi6p ........................G......................A...G...C........................

bi7 ................................................A..G............................

cd855 ................................................A..G....G................A......

cdsm ........................G......................A..GG...C........................

bi1 .................................................T.G............................

[ 11111111111111111111111111111111111111111111111111111111111111111111111111111111]

[ 22222222222222222222222222222222222222222222222222222222222222222222222222222222]

[ 00000000011111111112222222222333333333344444444445555555555666666666677777777778]

[ 12345678901234567890123456789012345678901234567890123456789012345678901234567890]

Ref AAAATAAACTTGCATCTCGAAGCTCAACGCTAAGCCCATGTAGCCCAGAGATATCTACAAGGCGTACCCTGAAGTAATAG

2004013 --..............................................................................

2004102 ......................................................................T.C.......

2004118 ..........................-...............................-.....................

2004163 .-.............................................................................A

2006439 .-........................................................-...........T.C.......

2007140 --.........-....................................................................

2007218 ................................................................................

2007825 ................................................................................

2007833 .--........--.....-..-...---............--.....---...-....---.--......-.-.......

2007837 ................................................................................

bi10 --....................-...--.............-.......-........-.....................

bi11 --....**GGTCCATTGTGATGGATGTGGTATGTCTTTTCGAAGATTTGAGTGCGGTAGTTGTATAGGTATC**....**CGGGC**.

bi13 .-....................................................................T.C.......

bi15 ......................................................................T.C.......

bi2 --.....-........................................................................

bi3 --..............................................................................

bi4 **TGGGCG**.................................................................G.A......

bi5 .-..............................................................................

bi6 --.........................-....................................................

bi6p .-..............................................................................

bi7 --..............................................................................

cd855 ......................................................................T.C.......

cdsm ................................................................................

bi1 ................................................................................

[ 11111111111111111111111111111111111111111111111111111111111111111111111111111111]

[ 22222222222222222223333333333333333333333333333333333333333333333333333333333333]

[ 88888888899999999990000000000111111111122222222223333333333444444444455555555556]

[ 12345678901234567890123456789012345678901234567890123456789012345678901234567890]

Ref ATGGTAGTCTTATACGGCTGCATATACAACCAGCGAAAATAGAACAGAAGGGACATCCTGATGAGAGTACGTCGATAAAA

2004013 ............................................................G...................

2004102 ..............................................................A.T...............

2004118 ............................................................G...................

2004163 ............................................................-...................

2006439 ...........................................-................-.A.T...............

2007140 .............................................-..............-..C.G..............

2007218 .......................................................-....-...................

2007825 ............................................................G...................

2007833 .....-.........-...-.....-.--.........-.--.-.--.......--....-..-.............--.

2007837 ..........................................................G.G...................

bi10 ........................................--.-.-..................................

bi11 **GCATACAGTCCGCGAATACAATAGCGTGTTTGTTAGGGGCCAGCTGAGGAAAG**.......-...................

bi13 .........................................................A..-.A.T...............

bi15 ................................................................T...............

bi2 ...........................................---..............-...................

bi3 .....................................................A......-.....A.............

bi4 .....................................................**ACCT**.........-**CGATGTAGAGGCG**

bi5 ................................................................................

bi6 .............................................-..............-...................

bi6p ...........................................................TG...................

bi7 ................................................................................

cd855 ..............................................................A.T...............

cdsm .............................................................C..................

bi1 ................................................................................

[ 11111111111111111111111111111111111111111111111111111111111111111111111111111111]

[ 33333333333333333333333333333333333333344444444444444444444444444444444444444444]

[ 66666666677777777778888888888999999999900000000001111111111222222222233333333334]

[ 12345678901234567890123456789012345678901234567890123456789012345678901234567890]

Ref AGTTCAATGGTATCCCTTCATGACTCTTAGCGTCGTCGATATCTGTGGAGTGGGTATACGATGATACCACAACCTTTCAG

2004013 .................---............................................................

2004102 ..................--............................................................

2004118 ..................--............................................................

2004163 ................................................................................

2006439 ..................--............................................-...............

2007140 .................---...........................................--...............

2007218 .................---............................................-...............

2007825 ..................--...........................................-................

2007833 ..-----......-...---..................................-........----....-....---.

2007837 ...................-............................................................

bi10 .....--..........---............................................................

bi11 ...............-...-............................................................

bi13 .................---............................................................

bi15 .............................--.................................................

bi2 .....--......-.--.--............................................................

bi3 ...................-............................................................

bi4 **GACCTGGCAACTCTTTCCTGGAGAGTACGATACTACTAGGGATCACAACACAAACGCGTTGGACCGATGTCGTTCCGAGA**

bi5 ..................--............................................................

bi6 ........-.........--............................................-...............

bi6p ................................................................................

bi7 ................................................................................

cd855 ................................................................................

cdsm ................................................................................

bi1 ................................................................................

[ 11111111111111111111111111111111111111111111111111111111111111111111111111111111]

[ 44444444444444444444444444444444444444444444444444444444444555555555555555555555]

[ 44444444455555555556666666666777777777788888888889999999999000000000011111111112]

[ 12345678901234567890123456789012345678901234567890123456789012345678901234567890]

Ref GATGGCTGTACGTCTAGCCGATCGGCGAAAGGTCGTTGATCTCTTGTCACGACATTCTGCGAAGATGAGTTTAGCTTTGA

2004013 ..--............................................................................

2004102 ..--....................................................-.......................

2004118 ..--....................................................-.......................

2004163 ..--....................................................-.......................

2006439 ..--....................................................--......................

2007140 ..--.........................-.........................--.......................

2007218 ..--....................................................-.......................

2007825 ..--.........................-..........................--......................

2007833 ----.--......---.......--..-.-..-.--.........-.....-------...--.......-....-.-..

2007837 ..--....................................................-.......................

bi10 ..--.......................-.-.......................-..-.......................

bi11 ..--...**ACGTACTCGATTAGCTAA**...............................-.......................

bi13 ..CC....................................................-.......................

bi15 T.CC....................................................-.......................

bi2 -............................-.......................--..-......................

bi3 .G..T.C......-...............-.........................--.......................

bi4 .G....C..................**TAGGGATCTAAGAGCTCAGCAATGTTGTGCCAGCTAGGAGCAGACCCGATCCCAG**

bi5 ...........................-....................................................

bi6 -....................................................-..........................

bi6p ................................................................................

bi7 ................................................................................

cd855 ................................................................................

cdsm ................................................................................

bi1 .....A..........................................................................

[ 11111111111111111111111111111111111111111111111111111111111111111111111111111111]

[ 55555555555555555555555555555555555555555555555555555555555555555555555555555556]

[ 22222222233333333334444444444555555555566666666667777777777888888888899999999990]

[ 12345678901234567890123456789012345678901234567890123456789012345678901234567890]

Ref GGAGCACCGTATGTCACTTCTATACCTGGCGTAGTCGTAGAAGAAGGGAACGCAGACCGGGTCAGTATGTCGATAAGCCA

2004013 ................................................................................

2004102 ................................................................................

2004118 ............................-...................................................

2004163 ..........................................................................-.....

2006439 ................................................................................

2007140 ............................-...................................................

2007218 ................................................................................

2007825 ................................................................................

2007833 --..----..-...-...-........---......-..........--.....-...............-..--.....

2007837 ................................................................................

bi10 ................................................................................

bi11 .....................................**CGAGGAGGAAAGGTATGAGTTAAACTCACGCAGTAGCGTCTTG**

bi13 ............................-...................................................

bi15 ............................-...................................................

bi2 ...........................---..................................................

bi3 ............................-...................................................

bi4 **AAGTTCTTTCCCACTGTGCACGCGTTGAATAGGAATA**...........................................

bi5 ................................................................................

bi6 ............................--............................................-.....

bi6p ................................................................................

bi7 ................................................................................

cd855 ................................................................................

cdsm ................................................................................

bi1 ................................................................................

[ 11111111111111111111111111111111111111111111111111111111111111111111111111111111]

[ 66666666666666666666666666666666666666666666666666666666666666666666666666666666]

[ 00000000011111111112222222222333333333344444444445555555555666666666677777777778]

[ 12345678901234567890123456789012345678901234567890123456789012345678901234567890]

Ref CGTTACAGCCACACCAATGACTCTTAGACGTTTTCCTGCTACGCTTTCAGGGGACGGGTTGACAGAAGGAAATGACTATT

2004013 .....................................................................G......-...

2004102 .....................................................................G......-C..

2004118 .....................................................................G.....--...

2004163 .....................................................................G......-...

2006439 .....................................................................G......-C..

2007140 .....................................................................G.....--...

2007218 .....................................................................G.....--...

2007825 .....................................................................G.....--...

2007833 .....-..................-...-................................-..-----G-....--...

2007837 .....................................................................G.....--...

bi10 ....................................................................-G.....--C..

bi11 **TACCGTGAAAGTGATGGCAGTCTCCCAGTACCCCTACATCCTATCCGTTAATAGAA**.**ACCAGTGATGAA**G**GGCAGT**-.**CC**

bi13 .....................................................................G.....--C..

bi15 ..........-..........................................................G......-C..

bi2 .....................................................................G.....--...

bi3 ............G...........................................C............G......-...

bi4 .........--.G........................-...............................G......-...

bi5 .....................................................................G......-...

bi6 .....................................................................G.....--...

bi6p .....................................................................G......-...

bi7 .....................................................................G......-C..

cd855 .....................................................................G..........

cdsm .....................................................................G..........

bi1 .....................................-......................................A...

[ 1111111111111111111111111111111111111111111111111111111111111]

[ 6666666666666666666777777777777777777777777777777777777777777]

[ 8888888889999999999000000000011111111112222222222333333333344]

[ 1234567890123456789012345678901234567890123456789012345678901]

Ref TTGGATGCCAGCCTACGCGCCGGGTCCTGCACCCTGTTTACCACACAATAATCTAACAGGG

2004013 .............................................................

2004102 .............................................................

2004118 .............................................................

2004163 .............................................................

2006439 .............................................................

2007140 .............................................................

2007218 .............................................................

2007825 .............................................................

2007833 ....--........-................-....-........-.--............

2007837 .............................................................

bi10 ..............................................-..............

bi11 **CCAACCAATGATACTTATAAAAAACTACATGTTTCACCCGATTTGACGCGGCTGG**......

bi13 .............................................................

bi15 .............................................................

bi2 ....................................-........................

bi3 ..........................................................A.A

bi4 ............................................-............G..A

bi5 .............................................................

bi6 ........................................................A....

bi6p .......................................................C.....

bi7 ........................................................A....

cd855 .............................................................

cdsm ...........................................................A.

bi1 .............................................................
