# Supplementary material for: The Impact of Recombination on dN/dS within Recently Emerged Bacterial Clones
Source: PLoS Pathog. 2011 Jul 14;7(7):e1002129. doi: 10.1371/journal.ppat.1002129 (PMC3136474; doi:10.1371/journal.ppat.1002129)
Supplement: Table S2 — dN/dS for 57 orthologous non-core genes in TW20 and MSHR1332. (DOC) [file ppat.1002129.s006.doc]

Supplementary Table S2

|  | dN/dS (as compared to ortholog in MSHR1332) |
| --- | --- |
| SATW20_00250 | 0.001 |
| SATW20_00360 | 0.1729 |
| SATW20_00370 | 0.035 |
| SATW20_01220 | 0.0296 |
| SATW20_03020 | 0.6416 |
| SATW20_03040 | 0.1143 |
| SATW20_03070 | 0.1401 |
| SATW20_03250 | 0.1161 |
| SATW20_03370 | 0.0073 |
| SATW20_03480 | 0.2177 |
| SATW20_03830 | 0.0378 |
| SATW20_04960 | 0.1808 |
| SATW20_04990 | 0.2124 |
| SATW20_06310 | 0.0671 |
| SATW20_06320 | 0.0819 |
| SATW20_06330 | 0.1006 |
| SATW20_08680 | 0.1083 |
| SATW20_08700 | 0.0209 |
| SATW20_08710 | 0.1244 |
| SATW20_08900 | 0.0177 |
| SATW20_11580 | 0.0827 |
| SATW20_13050 | 0.0896 |
| SATW20_13180 | 0.0388 |
| SATW20_13200 | 0.0133 |
| SATW20_13230 | 0.1262 |
| SATW20_14850 | 0.0728 |
| SATW20_18040 | 0.2103 |
| SATW20_19410 | 0.005 |
| SATW20_19430 | 0.0184 |
| SATW20_19440 | 0.0242 |
| SATW20_19450 | 0.087 |
| SATW20_19460 | 0.0886 |
| SATW20_19470 | 0.0447 |
| SATW20_19510 | 0.3998 |
| SATW20_19530 | 0.0522 |
| SATW20_19550 | 0.0968 |
| SATW20_19560 | 0.0319 |
| SATW20_19570 | 0.0239 |
| SATW20_19580 | 0.0048 |
| SATW20_19590 | 0.0627 |
| SATW20_19650 | 0.1215 |
| SATW20_19670 | 0.2213 |
| SATW20_19860 | 0.1114 |
| SATW20_19950 | 0.1968 |
| SATW20_19980 | 1.0325 |
| SATW20_20000 | 0.1728 |
| SATW20_20120 | 0.0048 |
| SATW20_20280 | 0.0301 |
| SATW20_20680 | 0.2651 |
| SATW20_20740 | 0.0131 |
| SATW20_26210 | 0.1075 |
| SATW20_26230 | 0.1007 |
| SATW20_28100 | 0.0197 |
| SATW20_28120 | 0.1105 |
| SATW20_28140 | 0.0811 |
| SATW20_28150 | 0.0426 |
| SATW20_28160 | 0.072 |
